# THE FOXO3 LONGEVITY GENOTYPE PROTECTS AGAINST MECHANISMS OF CELLULAR AGING IN OKINAWANS

**DOI:** 10.1093/geroni/igac059.1736

**Published:** 2022-12-20

**Authors:** Trevor Torioge, D Craig Willcox, Michio Shimabukuro, Timothy Donlon, Randi Chen, Richard Allsopp, Bradley Willcox

**Affiliations:** John A. Burns School of Medicine, University of Hawaii, Manoa, Honolulu, Hawaii, United States; Kuakini Medical Center, Honolulu, Hawaii, United States; Fukushima Medical University, Fukushima City, Fukushima, Japan; Kuakini Medical Center, Honolulu, Hawaii, United States; Kuakini Medical Center, Honolulu, Hawaii, United States; University of Hawaii, Honolulu, Hawaii, United States; Kuakini Medical Center, Honolulu, Hawaii, United States

## Abstract

The genetic association of FOXO3 genotypes with human longevity is well established, although the mechanism is not fully understood. We now report on the relationship of the FOXO3 longevity variant rs2802292 with telomere length, telomerase activity, FOXO3 expression and inflammatory cytokine levels in men and women. In agreement with earlier work, the FOXO3 longevity variant conferred protection against telomere shortening of peripheral blood mononuclear cells from adults aged 55 and older. This was accompanied by higher levels of telomerase activity in carriers of the longevity-associated FOXO3 G-allele (P=0.015). FOXO3 mRNA expression increased slightly with age in both young (P=0.02) and old (P=0.08) G-allele carriers. Older female G-allele carriers displayed a modest decline in levels of pro-inflammatory cytokine IL-6 with age (P=0.07). In contrast, older male G-allele carriers displayed an age-dependent increase in levels of anti-inflammatory cytokine IL-10 with age (P=0.04). Thus, FOXO3 may act through several different pro-longevity mechanisms, which may differ by age and sex.

